# Fabrication of nanochitosan incorporated polypyrrole/alginate conducting scaffold for neural tissue engineering

**DOI:** 10.1038/s41598-020-78650-2

**Published:** 2020-12-16

**Authors:** Asma Manzari-Tavakoli, Roghayeh Tarasi, Roya Sedghi, Ali Moghimi, Hassan Niknejad

**Affiliations:** 1 Department of Biology, Faculty of Science, Rayan Center for Neuroscience and Behavior, Ferdowsi University of Mashhad, Mashhad, Iran; 2 Department of Pharmacology, School of Medicine, Shahid Beheshti University of Medical Sciences, Tehran, Iran; 3 Department of Polymer and Materials Chemistry, Faculty of Chemistry and Petroleum Sciences, Shahid Beheshti University, G.C, 1983969411 Tehran, Iran

**Keywords:** Regenerative medicine, Tissue engineering

## Abstract

The utilization of conductive polymers for fabrication of neural scaffolds have attracted much interest because of providing a microenvironment which can imitate nerve tissues. In this study, polypyrrole (PPy)–alginate (Alg) composites were prepared using different percentages of alginate and pyrrole by oxidative polymerization method using FeCl_3_ as an oxidant and electrical conductivity of composites were measured by four probe method. In addition, chitosan-based nanoparticles were synthesized by ionic gelation method and after characterization merged into PPy–Alg composite in order to fabricate a conductive, hydrophilic, processable and stable scaffold. Physiochemical characterization of nanochitosan/PPy–Alg scaffold such as electrical conductivity, porosity, swelling and degradation was investigated. Moreover, cytotoxicity and proliferation were examined by culturing OLN-93 neural and human dermal fibroblasts cells on the Nanochitosan/PPy–Alg scaffold. Due to the high conductivity, the film with ratio 2:10 (PPy–Alg) was recognized more suitable for fabrication of the final scaffold. Results from FT-IR and SEM, evaluation of porosity, swelling and degradation, as well as viability and proliferation of OLN-93 neural and fibroblast cells confirmed cytocompatiblity of the Nanochitosan/PPy–Alg scaffold. Based on the features of the constructed scaffold, Nanochitosan/PPy–Alg scaffold can be a proper candidate for neural tissue engineering.

## Introduction

Neurodegenerative disorders such as the spinal cord and brain injury are a group of central nervous system (CNS) diseases that result from the loss of neurons and axons in the brain or spinal cord^1,2^. Since the central nervous system has little capacity to repair its damaged tissue, the regeneration of this tissue has many challenges^3,4^. Tissue engineering (TE) is a scientific approach to repair damaged tissues and organs, which has recently be focused on repairing or replacing lost neural tissues^5^. In TE, the scaffold provides a substrate for cell adhesion, differentiation and proliferation, which result in creation of a specific tissue with appropriate functions. Various types of biomaterials, either of natural or of synthetic origin, have been developed as inductive microenvironment for neural regeneration^6–8^. Among them, conductive polymers are very attractive candidates because of providing electrical signals which mimic native nerve tissues^9^. The electrically conductive-based scaffolds can be used for reconstruction of nerve, muscle and cardiac tissues^10^. Some conducting polymers including polyphenylene (PANI), polypyrrole (PPy), and polythiophene (PTh) have been extensively investigated for culture of electrical responsive cells. Among polymers, PPy has attracted particular attention in nerve regeneration because of its tunable electrical properties, biocompatibility, good environmental and thermal stability, easy synthesis, low toxicity and good mechanical properties^11–16^. It has been reported that polypyrrole-based scaffolds can augment adhesion and growth of wide range of cells such as neuronal cells, glia cells derived from dorsal root ganglia, endothelial and mesenchymal cells. Moreover, implanted PPy showed neuron regeneration and axon growth with minimal immune response in vivo^17^. One of the limitations of using conductive polymers is their intrinsic inability to degrade in vivo, which maybe causes induction of chronic inflammation. Furthermore, the fragility and incapability to mechanically manipulation and processing make it difficult to use conductive polymer alone. Therefore, the majority of studies have focused on biological and physical modification to construct composite based conductive polymers. Biocompatibility and biodegradation property of conductive polymers is generally achieved by modification them with suitable natural biodegradable polymers such as alginate^12,16,18,19^.

Sodium alginate (SA) is a natural biocompatible hydrophilic polysaccharide. It is non-toxic and non-immunogenic polymer, which resemble the extracellular matrix of the body. SA is biodegradable linear carbohydrate biopolymers derived from brown algae. It has been shown that alginate-based scaffolds improve cell adhesion and cell proliferation which result in repairing some organs including skin, nerve and liver^9,20^. Alginate-based scaffolds were also used as an appropriate substrate for construction of conductive scaffolds based on pyrrole monomers using electrostatic interactions between carboxylate moieties of alginate and positively charged PPy^16^. Several studies have designed alginate-pyrrole based scaffolds or hydrogels with electrical properties for neural tissue engineering^21–23^.

Chitosan as a natural polysaccharide and its improved derivatives have frequently been used in tissue engineering and regenerative medicine^24^. It has been shown that chitosan incorporated alginate-pyrrole scaffold provides an interactive substrate between the seeded cells and external electric field^18^. In recent years, many studies investigated the roles and biological functions of nanoparticles in the scaffold^25^. Nanoparticles possess exclusive properties such as large surface-to-volume ratio and high surface reactivity which make them as suitable substrate for adhesion and proliferation of various cells. Chitosan nanoparticles are a natural biomaterial with many advantages such as biodegradability, proper biocompatibility and being a controlled-release carrier for growth factor delivery^24,26^. Also, the use of the nanochitosan in the scaffold increases the specific surface area and surface energy which makes the scaffold more hydrophilic. The hydrophilic surface of scaffold is favorable for attachment of the cells to the scaffolds^27,28^. Chitosan nanoparticles can be crosslinked by interactions of negatively charged sodium tripolyphosphate (TPP) with positively functional groups of Chitosan^26^.

Since previous studies have reported that the amount of conductive polymers can affect the electrical properties of conductive scaffold, we hypothesized here that the application of different percentages of alginate and pyrrole affects characteristics of the electrical conductive scaffolds. Therefore, the first aim of this study was to evaluate the effects of different percentages of alginate and pyrrole on the electrical conductivity properties of PPy–Alg composites in order to find the optimal concentration. As second goal to improve the hydrophilicity of the PPy–Alg composite, nanochitosan was synthesized and characterized and then integrated with PPy–Alg polymers blend, in order to construct a novel polypyrrole based conducting scaffold with proper physiochemical characterizations for neural tissue engineering.

## Materials and methods

### Preparation of polypyrrole–alginate composites

**Figure 1:**
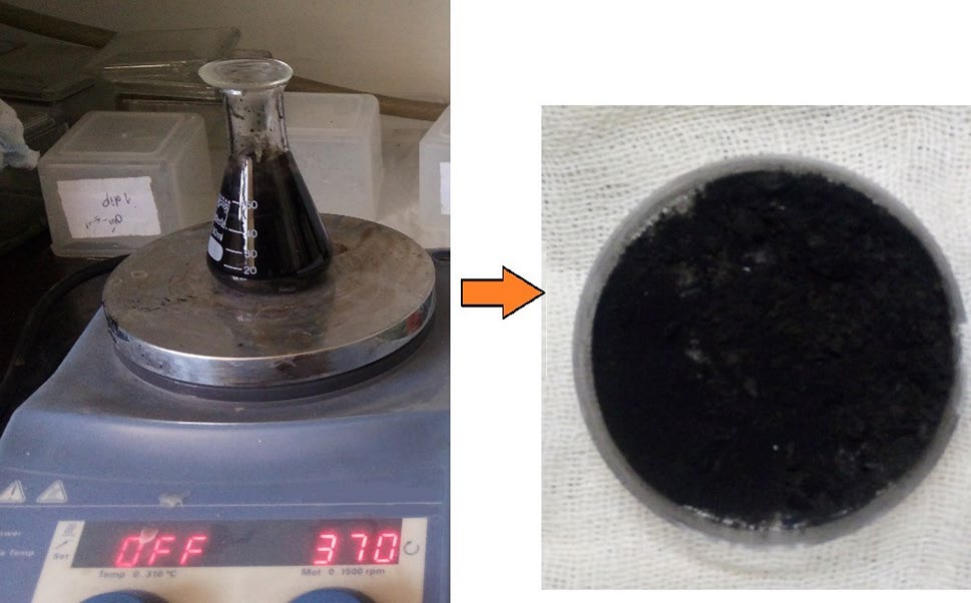
Representative image for synthesis of polypyrrole–alginate composite.

Pyrrole (0.1 M, sigma) solution was made using HCl (1 N) and was added drop by drop to alginate solution (3%, sigma) with volume ratios 1: 10 and 2: 10 and stirred for 30 min at 90 °C. Then, ferric chloride (FeCl_3_, 0.2 M) was added slowly to this solution till its color changed to black within 5 h stirring^18^. The polymer mixtures were isolated from the reaction mixture using a dialysis membrane with cut off 12–14 kDa (D9402, Sigma) for 5 days to eliminate non-reactive and oxidant substances. The polymer was put in freeze dryer to dry out for 24 h (Fig. 1).

### Synthesis of nanochitosan

Nanochitosan were synthesized as we described in our previous study with some modifications^27^. Briefly, chitosan (0.9 mg/ml, Medium molecular weight chitosan, Mw = 50,000–190,000, Sigma) was dissolved in 5 ml acetic acid 0.5% and was stirred for 24 h until a homogeneous solution is obtained. Then, NaOH (2 M) is added until its pH was adjusted to 5.5. Two milliliters TPP solution (0.25 mg/ml, Sigma) was added drop wise to the chitosan solution with rate (0.2 ml/min) and stirred for 1 h to form nanochitosan. In order to measure size and polydispersity index (PdI) of synthesized nanochitosan and their morphology, dynamic light scattering (DLS) and SEM were carried out, respectively.

### Fabrication of nanochitosan/polypyrrole-alginate scaffold

Initially, 3gr of nanochitosan powder was dispersed in 100 ml acetic acid (1%) and was stirred for 30 min. one gram powdered PPy–Alg (2:10) was added to this mixture and again stirred for 5 h. Finally, the samples were put in freeze dryer to dry out for 24 h and then cross-linked with glutaraldehyde (0.25%) for 12 h. The scaffolds were further washed with PBS for 24 h and again dried with a freeze dryer.

## Characterization

### Characterization of PPy–Alg composites

The chemical structure of PPy–Alg composites was evaluated by FTIR spectrum, which was registered on a Thermo Nicolet Nexus 470 Fourier transform infrared spectrometer in the domain of 650–4000 cm^−1^. In order to measure the electrical conductivity, PPy–Alg composites with volume ratios of 1:10 and 2: 10 were casted separately to form film. Electrical conductivity of the produced PPy–Alg films was measured by four-probe technique (4point probe measurement, 4PP-R2K). The conductivity was calculated using the following formula (Eqs. 1 and 2)^29^:

1 $$Resistivity\left( {\rho , ohm - cm} \right) = {\raise0.7ex\hbox{${\pi t}$} \!\mathord{\left/ {\vphantom {{\pi t} {ln2}}}\right.\kern-\nulldelimiterspace} \!\lower0.7ex\hbox{${ln2}$}}\left( {{\raise0.7ex\hbox{$V$} \!\mathord{\left/ {\vphantom {V I}}\right.\kern-\nulldelimiterspace} \!\lower0.7ex\hbox{$I$}}} \right) = 4.53 \times t \times \left( {resistance} \right)$$

2 $$Conductivity \left( {{\raise0.7ex\hbox{${\sigma ,S}$} \!\mathord{\left/ {\vphantom {{\sigma ,S} {cm}}}\right.\kern-\nulldelimiterspace} \!\lower0.7ex\hbox{${cm}$}}} \right) = {\raise0.7ex\hbox{$1$} \!\mathord{\left/ {\vphantom {1 \rho }}\right.\kern-\nulldelimiterspace} \!\lower0.7ex\hbox{$\rho $}}$$

where $resistance$ is the amount of resistance measured by the device, *V* is measured voltage, *I* is applied current and *t* is the thickness of the sample.

### Characterization of nanochitosan/PPy–Alg Scaffold

#### Electrical conductivity measurement

The amount of electrical conductivity of the produced nanochitosan/PPy–Alg scaffold was evaluated by four-probe technique. The Eqs. (1) and (2) (as mentioned above) were used to calculate the conductivity.

#### Scanning electron microscopy (SEM)

The porous structure and morphology of scaffold and nanochitosan were analyzed through SEM instrument (Jeol, Tokyo, Japan), as described previously^30^.

#### Water contact angle test

The surface wettability of PPy–Alg composite and nanochitosan/PPy–Alg scaffold was investigated by measuring contact angle. This was done by placing a drop of water on the composite and scaffold surface and the contact angle was evaluated by OCA PLUS 15 device.

#### Porosity evaluation

The liquid displacement method was carried out to measure porosity of the nanochitosan/PPy–Alg scaffold. Pre-weighed scaffold was submerged in a determined volume of absolute ethanol for 48 h. Then, the ethanol saturated scaffold was removed and was weighed again. Each experiment was carried out 3 times and the average of the porosity of scaffold was obtained. The porosity was measured using the following formula (Eq. 3)^30^:

3 $$Porosity\left( \% \right) = \left( {V2 - V1 - V3} \right)/\left( {V2 - V3} \right) \times 100$$

V1, the initial weight of the scaffold; V2, total weight of ethanol and immersed scaffold; V3, the weight of ethanol after removing scaffold.

#### Swelling evaluation

The water uptake ability of the nanochitosan/PPy–Alg scaffold was evaluated by immersing scaffold in PBS (pH = 7.4) for different time periods (1, 7, 14, 21 days) at 37 °C. After the mentioned time intervals, scaffolds were rinsed with deionized water and the surface water was removed using filter paper and samples were weighed (Wet weight). Experiments were done in triplicate. The swelling ratio was calculated using the following formula (Eq. 4)^31^:

4 $$Swelling \,ratio = Wet \,weight - Dry \,weight/Dry \,weight$$

#### Degradation measurement

The scaffold was weighed (WI) and then it was immersed in PBS (1x, pH = 7.4) and incubated at 37 °C for different time intervals (1, 7, 14, 21 days). After completion of a predetermined time period, the scaffold rinsed with distilled water, dried at 37 °C for 24 h in an oven and weighed again (Wt). Degradation (%) was determined using the following formula (Eq. 5):

5 $$Degradatin \left( \% \right) = \left( {WI - Wt} \right)/WI \times 100$$

## Cell culture study

OLN-93 neural cell line (rat brain neural cells) and Normal human dermal fibroblasts (NHDF) were purchased from Pasteur Institute. The cells were cultured in flask (25 cm^2^) with Dulbecco’s Modified Eagle Medium (DMEM) containing 10% Fetal Bovine Serum (FBS) and 1% penicillin–streptomycin and incubated in an incubator at 37 °C containing 5% CO_2_ and 95% air. The media was exchanged every 3 days. The experimental procedures in this study were approved by Ethic Research Committee of Shahid Beheshti University of Medical Sciences under the ethical code number of IR.NIMAD.REC.1398.388. All methods were performed in accordance with the relevant guidelines and regulations of Shahid Beheshti University of Medical Sciences.

### Study of cytotoxicity and proliferation of scaffold

The cytotoxicity and proliferation of nanochitosan/PPy–Alg scaffold was assessed by the colorimetric MTT assay. The scaffolds for the cell culture were sterilized with UV radiation from both sides for 30 min each, and then incubated for 24 h in DMEM to increase the adhesion of the cells. On the next day, media removed and scaffolds were placed in 96-well plate, then OLN-93 neural cells and fibroblast cells were detached using trypsin–EDTA (0.15%) and 10,000 cells per well were seeded on each scaffold. 150 μl complete cell culture medium was added to each well and incubated at 37 °C. A group of cells were seeded in tissue culture plate and supplemented with complete DMEM medium which was evaluated as the control group. In this study, we used MTT assay to measure cytotoxicity after 24, 48 and 72 h of culture. Also, to assess the proliferation of OLN-93 neural cells, cell viability was evaluated after 7 and 14 days of culture by MTT assay. For this aim, MTT solution (15 μL, 5 mg/ml) was added to each well at mentioned time intervals and then the cell culture plates incubated for 4 h. Following the incubation, the media was replaced with 150 μL of DMSO to dissolve the formazan crystals for 15 min. After removing the scaffold from each well, the optical absorption was measured by an ELISA reader (BioTek, USA) at 570 nm.

### Study of adhesion of OLN-93 neural cells on scaffold

The scaffold was sterilized using UV, and then incubated for 24 h in complete cell culture medium. The next day, medium completely removed and OLN-93 neural cells were seeded on the scaffold and again incubated at 37 °C for 24 h. Subsequently, glutaraldehyde (2.5%) was used for fixation of the cells on the scaffold for 2 h and dehydrated with different concentrations (50, 70, 90, and 100%) of alcohol^18^. Finally, morphology and elongation of OLN-93 neural cells were observed using SEM.

### Histology study

Histological studies were carried out to investigate cell distribution on the nanochitosan/PPy–Alg scaffold. The scaffolds were sterilized using UV, and then incubated for 24 h in DMEM. The next day, medium was completely removed and OLN-93 neural cells were cultured on the scaffold and incubated in an incubator at 37 °C containing 5% CO_2_ and 95% air for 24 h. Then glutaraldehyde (2.5%) was used for fixation of the cells for 2 h and dehydrated with different concentrations (50, 70, 90, and 100% v = v) of alcohol for histological assay. Afterwards, the scaffolds seeded with the cells were embedded in paraffin by standard techniques. Then the sections of 4 µm thickness were cut with a microtome (MICRO DS, 4055) and were stained with hematoxylin and eosin (H&E) technique for light microscope investigation.

### Statistical analysis

All data were presented as means ± SD. Analysis of variance (ANOVA) was used with Turkey's post-test to determine statistical significance. A *p* value of < 0.05 was considered to be statistically significant.

## Results and discussion

### Fourier transform-infrared spectroscopy

**Figure 2:**
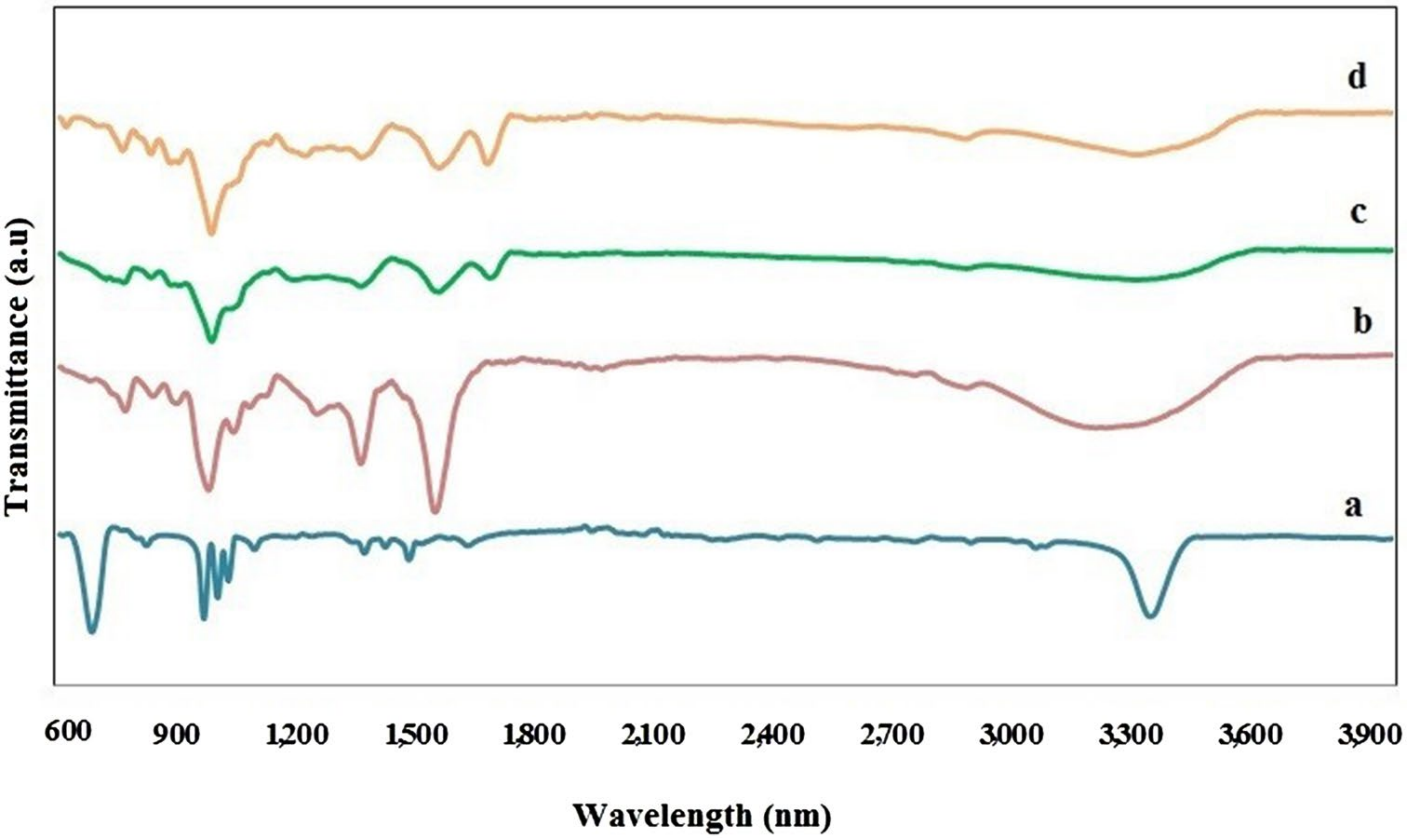
FTIR spectra of (**a**) pyrrole, (**b**) alginate, (**c**) alginate 3% + 1 cc pyrrole, (**d**) alginate 3% + 2 cc pyrrole.

PPy–Alg composites were produced by chemical polymerization of the pyrrole monomer using FeCl_3_ as an oxidant agent. Pyrrole monomers was allowed to react with alginate in acidic condition. Then, polymerization was initiated by adding FeCl_3_. It was put in freeze dryer for 24 h to form PPy–Alg Composites powder is illustrated in Fig. 1. FTIR spectrum analysis of pyrrole, alginate and PPy–Alg composites have shown in Fig. 2. The spectrum of FTIR obtained for pyrrole indicates the presence of characteristic absorption peaks of C=C (stretching of pyrrole ring) at 1550 cm^−1^, C=N at 1427 cm^−1^, N–H at 3391 cm^−1^, and =C–H (pyrrole ring) at 3130 and 3102 cm^−1^. Further, the weak peak at 2849 cm^−1^ is due to C-H stretching. Alginate displays its characteristic bands at 3279, 2932 and 1620 cm^−1^ of O–H, C–H and C=O, respectively. The presence of two bands at 1594 and 1464 cm^−1^ of PPy in the FTIR spectrum of PPy–Alg composite, confirms the presence of PPy in the two composites of pyrrole (1 cc)-Alginate 3% and pyrrole (2 cc)-Alginate 3%. The intensity of peaks of alginate at 3500 have been decreased strongly in two composites compared with pure alginate. The absorption band at 1620 cm-^−1^ allocated the peak of the carbonyl bond peak in the pure alginate spectrum shifted to a higher wavelength (1700 cm^−1^) which is likely due to the complete interaction between alginate and PPy.

### Electrical conductivity

Measurement of the electrical conductivity of films produced from PPy–Alg composites and nanochitosan/polypyrrole-alginate scaffold has been performed by four-probe apparatus at room temperature. The calculated electrical conductivity values are shown in Table 1. It is obvious that the films conductivity increased by increasing the amount of pyrrole. The composites with ratio 2: 10 of pyrrole and alginate possess higher conductivity. It has been reported that the amount of conductive polymers can impress the electrical properties of conductive scaffolds^21^. Based on this result, polymers with 2:10 ratio of pyrrole and alginate was determined to incorporate with chitosan nanoparticles to synthesize the final scaffold. In this study, we used concentrations higher than 2 ml of 0.1 M pyrrole in our pilot studies. The films made from compositions with concentrations higher than 2 ml of PPy were fragile, so they were broken to pieces during the drying process or within evaluation of their surfaces by four-point probe. These results were consistent with previous studies in which showed more PPy concentration more fragility of film^10,21,22^. As a result, the composite ratio 2:10 was chosen which provides a suitable mechanical property and electrical conductivity.

**Table 1:** Conductivity measurements of PPy–Alg composites.

Polymer	Conductivity (σ S/cm) ± SD
Pyrrole 0.1 M:Alginate3% (1:10)	0.0002 ± 0.00004
Pyrrole 0.1 M:Alginate3% (2:10)	0.001 ± 0.0001

### Morphology and size of nanochitosan

**Figure 3:**
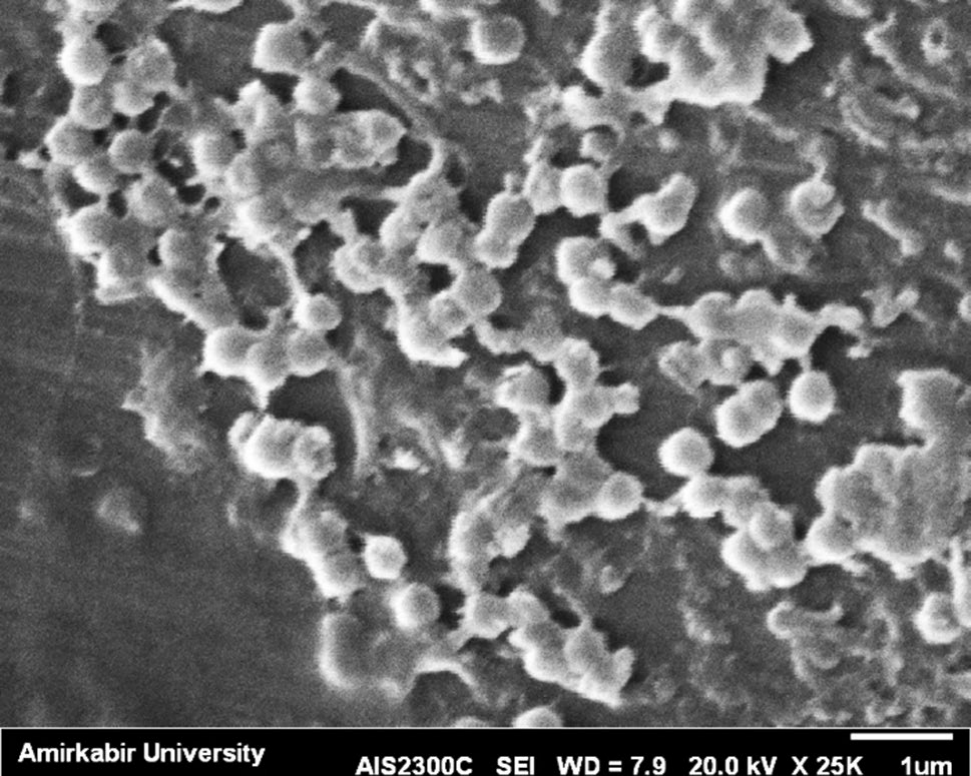
SEM image of chitosan nanoparticles.

Nanochitosan particles were usually synthesized by the iontropic gelation method. Nanochitosan particles were created by the electrostatic interaction between the protonated amine groups in chitosan and the polyanion sodium tripolyphosphate (TPP). The DLS results for synthesized nanochitosan particles is indicated that their distribution was in the range of 39–243 nm (PdI = 0.234). SEM image of nanochitosan particles in Fig. 3 shows that nanoparticles were spherical and clustered shape. These result demonstrated that the chitosan nanoparticles mostly distributed in nano size. It has been shown that the nano size of chitosan demonstrate better results in cell growth and nanochitosan incorporated scaffolds provide a superior support for tissue regeneration^32^. Narrow size distribution of PdI (near 0.2) demonstrates a low tendency to accumulate^33^.

### Morphology and porosity of nanochitosan/PPy–Alg scaffold

**Figure 4:**
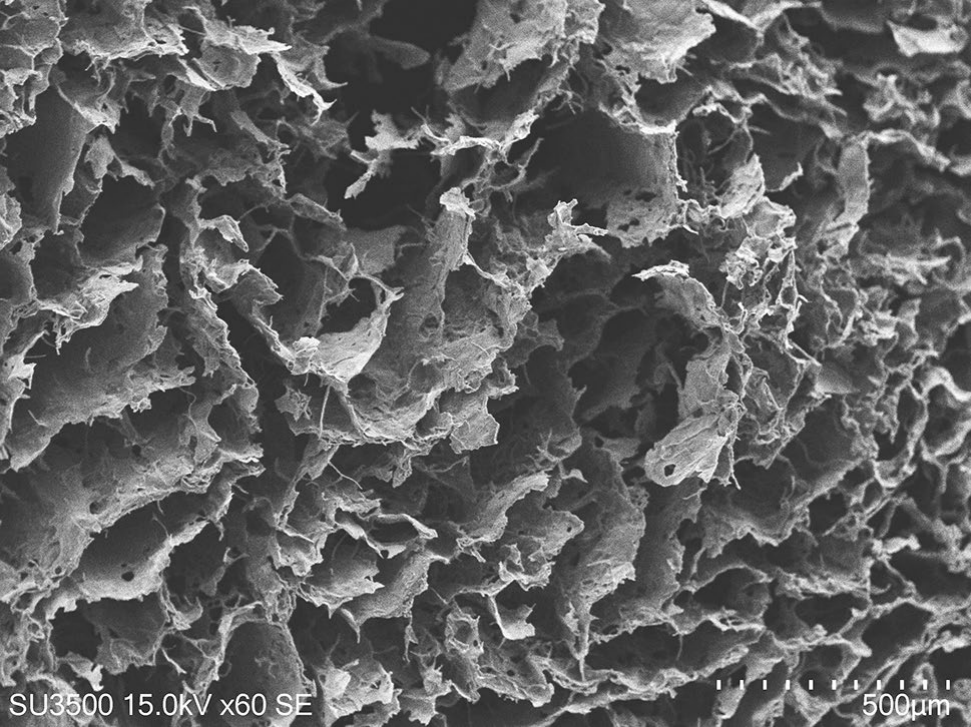
The SEM image shows the morphology of nanochitosan/PPy–Alg scaffold which is prepared with ratio 2:10 polypyrrole–alginate composite.

Designing a proper scaffold for neural tissue engineering is crucial for the successful regeneration of tissue. Therefore, electrically conductive polymers are promising substrates in manufacturing of scaffolds for neural tissue engineering^9,34^. In this study, nanochitosan particles were incorporated into PPy–Alg (2:10) composite for obtaining a hydrophilic, processable and stable scaffold. The results showed that incorporation of nanochitosan into PPy–Alg composite did not change the electrical conductivity and the nanochitosan/PPy–Alg scaffold possesses electrical conductivity. SEM images showed that the nanochitosan/PPy–Alg scaffold has a clear porous structure (Fig. 4). The porous structure of nanochitosan/PPy–Alg scaffold can improve cell adhesion, enhance delivery of nutrients, mediums and soluble signaling molecules to the seeded cells, and also provide a condition for metabolic waste removal. Hence, it can be used as a scaffold for tissue engineering applications. In addition, the porosity of the prepared scaffold was evaluated based on Eq. (3). The scaffold exhibited a porosity of about 97.17 ± 0.14%. Total porosity greater than 90% is optimal for polymeric scaffolds to be used in tissue engineering and can allow the cells to migrate into the scaffolds^30^.

### Scaffolds’ wettability measurements

The water contact angle values of PPy–Alg composite was 96.7 which shows that PPy–Alg composite is not a proper hydrophilic structure; while, incorporating chitosan nanoparticles into the PPy–Alg structure make it completely hydrophilic. Since the wettability of the nanochitosan/PPy-Alg scaffold was very higher than the produced PPy–Alg composite, we showed the wettability (water absorption) in a video (Supplementary Video, Media 1). It seems that the use of the nanochitosan in the scaffold increases the specific surface area and surface energy and makes the scaffold more hydrophilic. Consistent to this result, the other studies have reported that due to hydrophilic groups which present on nanochitosan surface, its incorporation increases the absorption of water^27,28^.

### Swelling evaluation

**Figure 5:**
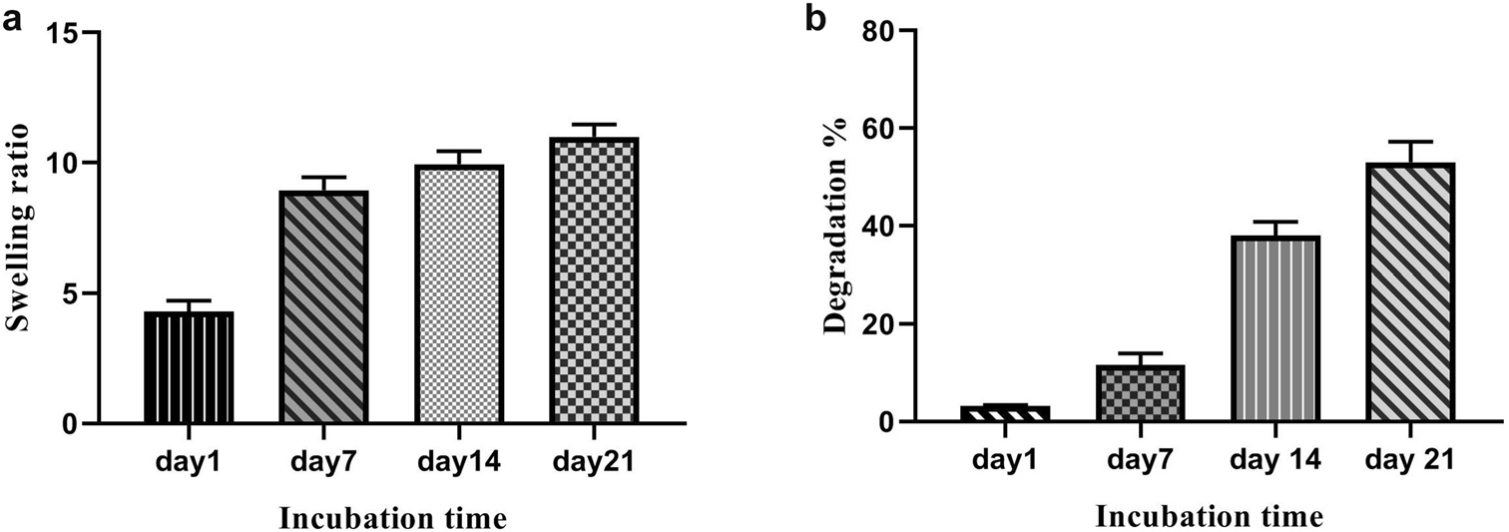
The effects of incorporating nanochitosan into the PPy–Alg composite on swelling and degradation the scaffold: (**a**) swelling ratio for 1, 7, 14, 21 days, (**b**) degradation study of scaffold for 1, 7, 14, 21 days.

Water uptake behavior of the scaffold was measured in PBS solution at 37 °C. Swelling of the scaffolds involves the uptake of body fluids which facilitates the transfer of nutrients and causes cellular penetration into the scaffold^31^. As shown in Fig. 5a, the results of the swelling test indicated that nanochitosan/PPy–Alg scaffold increased the swelling during the time period of 21 days, which is appropriate for further cell adhesion and cell penetration into the scaffold.

### Degradation measurement

Degradation of nanochitosan/PPy–Alg scaffold by incubating in the physiological condition (PBS; 37 °C) was examined for 21 days. As shown in Fig. 5b, the weight loss ratio of scaffolds enhanced progressively over the entire 21-day period. The results showed that the degradation of scaffold had gradual enhancement for different time periods (1, 7, 14, 21 days). This controlled degradation is a critical factor in tissue engineering which can be related to the cross-linkers used in the construction of the scaffold.

### Study of cytotoxicity and proliferation

**Figure 6:**
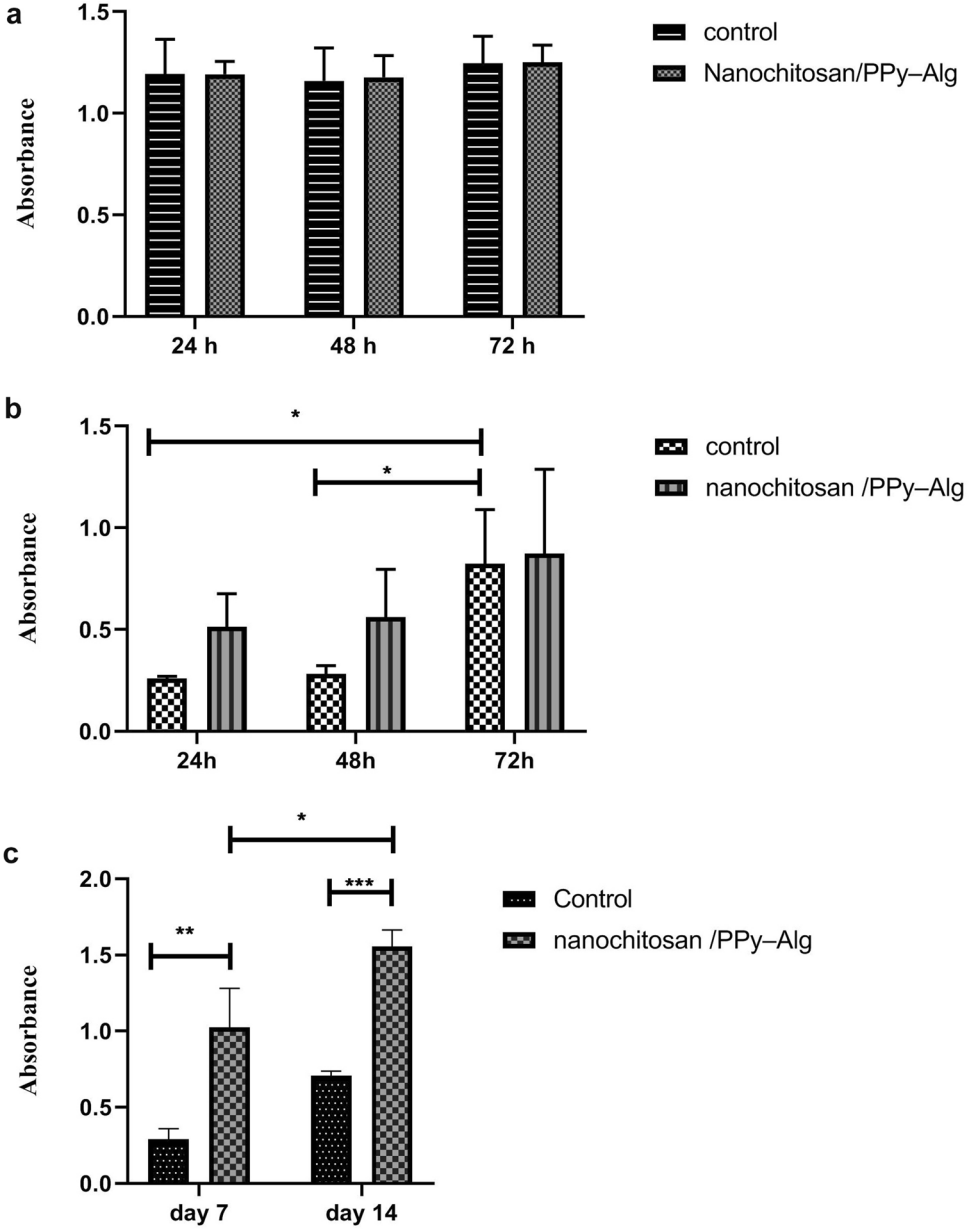
(**a**) Cytotoxity of fibroblast cells seeded on nanochitosan/PPy–Alg scaffolds after 24, 48 and 72 h by MTT assay. (**b**) Cytotoxity of OLN-93 neural cells cultured on nanochitosan/PPy–Alg scaffolds after 24, 48 and 72 h by MTT assay. (**c**) The results of proliferation of OLN-93 cells cultured on nanochitosan/PPy–Alg scaffold after 7 and 14 days. *: *p* < 0.05, **: *p* < 0.001 and ***: *p* < 0.0001.

Viability of OLN-93 neural cells and normal fibroblast cells was assessed by MTT assay in days 1, 2, 3, 7 and 14 after culture. Figure 6a,b show cell viability of fibroblast and OLN-93 neural cells in nanochitosan/PPy–Alg scaffold, after 24, 48 and 72 h of cell culture, respectively. These results demonstrate that nanochitosan/PPy–Alg scaffold did not induce any cytotoxic effects on fibroblast cells and OLN-93 neural cells. The proliferation of OLN-93 cells on nanochitosan/PPy–Alg scaffold was examined using MTT assay after the cells were seeded on the scaffold for 7 and 14 days. As shown in Fig. 6c, the proliferation results of OLN-93 cells demonstrated that the number of cells significantly increased from day 7 to 14 which might be due to proliferation of the cells. Overall we concluded that the prepared scaffold is biocompatible and non-cytotoxic.

### Study of adhesion of OLN-93 neural cells

**Figure 7:**
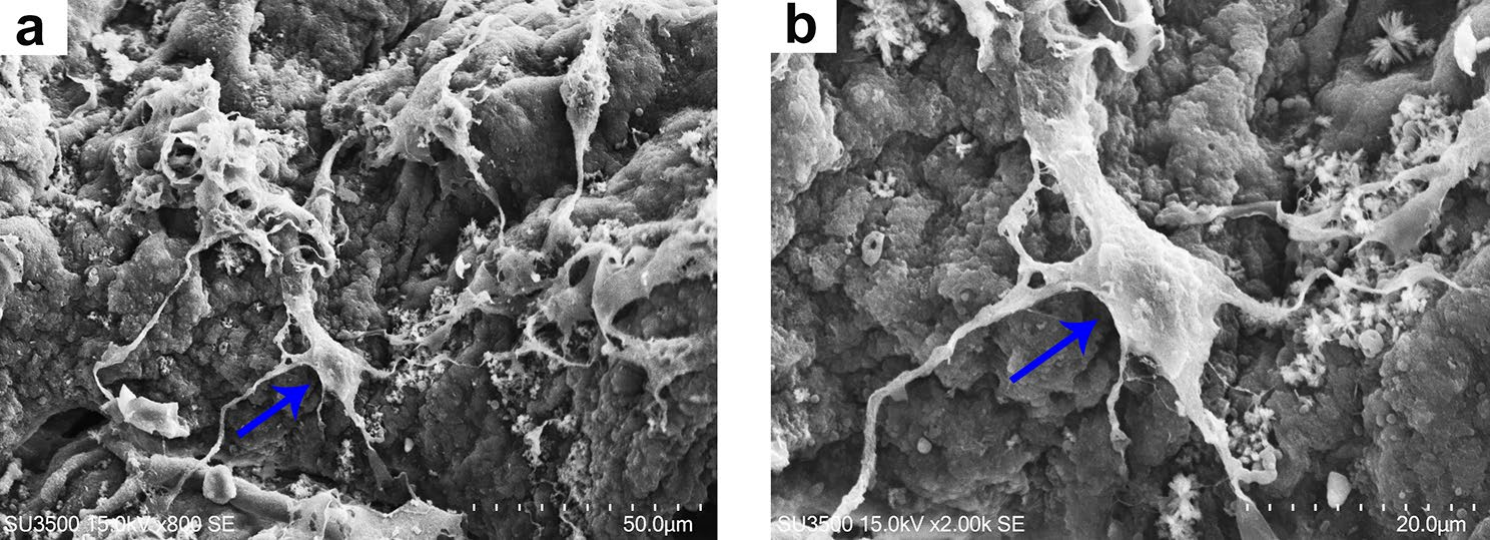
(**a**) The attachment of OLN-93 neural cells on nanochitosan/PPy–Alg scaffold. (**b**) High magnitude illustration of the cell which shows by blue arrow.

SEM images shows that the OLN-93 neural cells were attached well on the nanochitosan/PPy–Alg scaffold surface after 24 h (Fig. 7). These results showed that nanochitosan/PPy–Alg scaffold promoted OLN-93 neural cells adhesion and the cells expressed their characteristic morphology. Previous studies have shown that the surface characteristics of scaffolds significantly affect cell adhesion and hydrophilic surface of scaffolds were favorable for attachment of the cells to scaffolds^35^. Moreover, chitosan nanoparticles in scaffolds probably have a role in nanoparticles-dependent attachment via involving in the cell membrane.

### Histology studies

**Figure 8:**
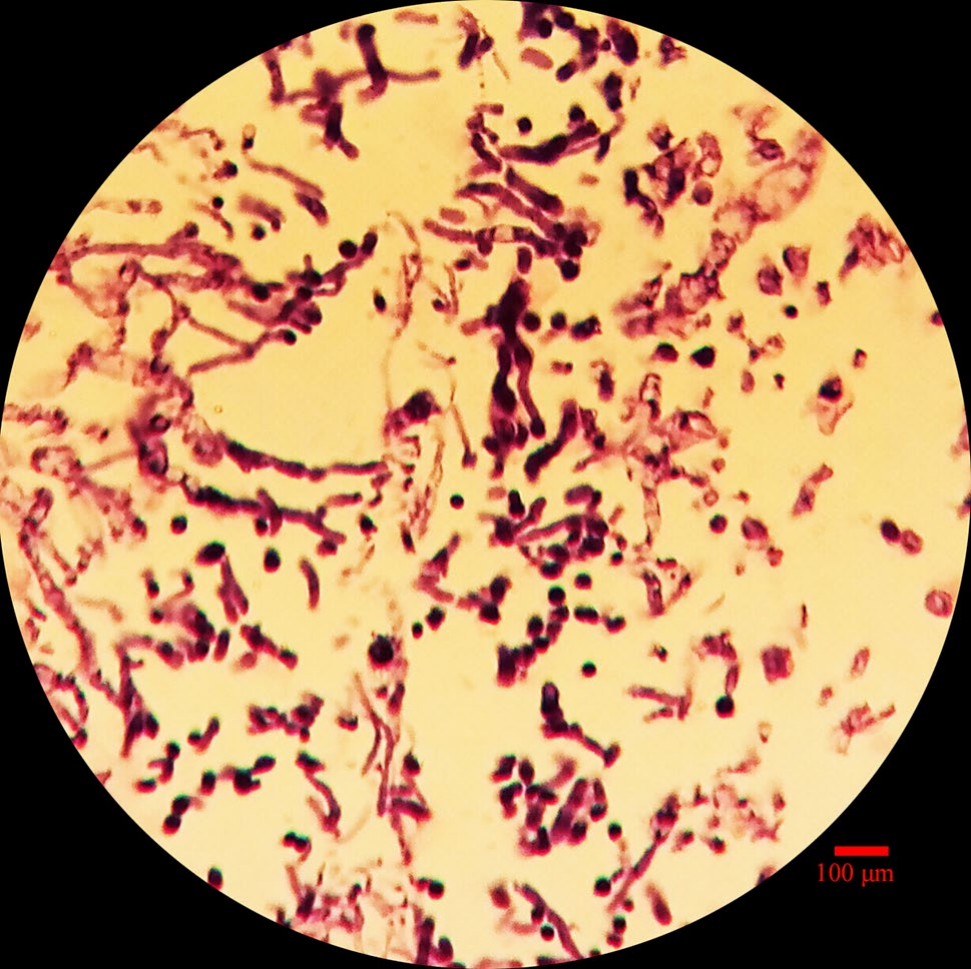
H&E staining optical micrograph of OLN-93 neural cells cultured on Nanochitosan/PPy–Alg scaffold for 24 h.

Figure 8 shows the optical image of OLN-93 with uniform distribution of the cells. The nanochitosan/PPy–Alg scaffolds were stained with H&E in which cell nuclei appeared in purple and scaffold in red. The histological results showed that the neural cells attached well to the scaffold and no cell cluster was obvious in the figure.

## Conclusion

Polypyrrole–alginate composites were synthesized with different wt% of pyrrole and alginate. The results show PPy–Alg composite with ratio 2:10 possesses high conductivity which served as a suitable material, which was incorporated with nanochitosan to fabricate nanochitosan/PPy–Alg scaffold for tissue engineering applications. The cytocompatibility and cell attachment of scaffold were evaluated using OLN-93 and fibroblast cells. It can be concluded nanochitosan/PPy–Alg scaffold has proper conductivity and capability for neural cell attachment and proliferation. The future research would be required to evaluate this conducting scaffold for neurodegenerative disorders such as spinal cord injuries in in vivo studies.

## Supplementary Information

Supplementary Information.

Supplementary Video.

## Data Availability

All data are available in this study.
