# Supplementary material for: Fabrication of nanochitosan incorporated polypyrrole/alginate conducting scaffold for neural tissue engineering
Source: Sci Rep. 2020 Dec 16;10:22012. doi: 10.1038/s41598-020-78650-2 (PMC7744540; doi:10.1038/s41598-020-78650-2)
Supplement: Supplementary file 1 — Supplementary Information. [file 41598_2020_78650_MOESM1_ESM.docx]

**Fabrication of Nanochitosan Incorporated Polypyrrole/Alginate Conducting Scaffold for Neural Tissue Engineering**

Asma Manzari-Tavakoli^1^, Roghayeh Tarasi^2^, Roya Sedghi^3^, Ali Moghimi*^1^, Hassan Niknejad*^2^

^1^Rayan Center for Neuroscience & Behavior, Department of Biology, Faculty of Science, Ferdowsi University of Mashhad, Iran.

^2^ Department of Pharmacology, School of Medicine, Shahid Beheshti University of Medical Sciences, Tehran, Iran.

^3^ Department of Polymer & Materials Chemistry, Faculty of Chemistry and Petroleum Science, Shahid Beheshti University, G.C, 1983969411, Tehran, Iran.

* Corresponding authors:

Department of Pharmacology, School of Medicine, Koodakyar St, Deneshjoo Blvd, Velenjak, Tehran, Iran.

P.O. Box 1985717446,

E-mail address: [niknejad@sbmu.ac.ir](mailto:niknejad@sbmu.ac.ir)

^1^Rayan Center for Neuroscience & Behavior, Department of Biology, Faculty of Science, Ferdowsi University of Mashhad, Iran.

E-mail address: [moghimi@um.ac.ir](mailto:moghimi@um.ac.ir)

**Video Legend**

The comparison of wettability (water absorption) between the nanochitosan/PPy-Alg scaffold and PPy-Alg composite.
